# Topiramate-induced severe heatstroke in an adult patient: a case report

**DOI:** 10.1186/s13256-016-0835-5

**Published:** 2016-04-13

**Authors:** Lucie Canel, Sofia Zisimopoulou, Marie Besson, Mathieu Nendaz

**Affiliations:** 1 Department of Internal Medicine, HUG Geneva University Hospitals, Rue Gabrielle-Perret-Gentil 4, 1205 Geneva, Switzerland; 2 Department of Community Medicine, HUG Geneva University Hospitals, Rue Gabrielle-Perret-Gentil 4, 1205 Geneva, Switzerland; 3 Department of Clinical Pharmacology and Toxicology, HUG Geneva University Hospitals, Rue Gabrielle-Perret-Gentil 4, 1205 Geneva, Switzerland

**Keywords:** Topiramate, Heatstroke, Adult, Anhidrosis, Febrile coma

## Abstract

**Background:**

Heatstroke is a life-threatening condition defined by failure of heat load dissipation, resulting in a core temperature higher than 40 °C (104 °F) associated with neurological dysfunction. Topiramate may cause anhidrosis, potentially resulting in heatstroke, as reported especially in children.

**Case presentation:**

A 57-year-old Caucasian man was admitted to the emergency room in a febrile comatose state. After a complete workup ruling out the usual etiologies of such a condition, we assumed the hypothesis of a heatstroke caused by topiramate, recently prescribed for essential tremor.

**Conclusions:**

Topiramate-related heatstroke has been described in children but must be recognized in adults as well. Outcomes may range from total clinical recovery to persistent neurological dysfunction or death. The prescription of topiramate and the follow-up of adult patients under this medication should include an evaluation of hypohidrosis, especially in contexts of high temperature.

## Background

Heatstroke is a life-threatening condition defined by failure of heat load dissipation, resulting in a core temperature higher that 40 °C (104 °F) associated with neurological dysfunction, ranging from mild altered mental status to coma [1]. Mortality is high among patients presenting to the hospital. Two types can be distinguished, exertional and classical heatstroke. Exertional heatstroke is related to strenuous exercise during periods of high temperature. Classical heatstroke is favored by any mechanism decreasing heat loss (anticholinergic or antihistamine drugs, advanced age, high ambient temperature,…) or increasing heat production (sepsis, stimulant drugs, thyroid storm,…). Multiple organ complications are common, including acute respiratory distress syndrome, acute renal failure, hepatic injuries, and rhabdomyolysis.

Topiramate has been described as a trigger for heatstroke, essentially in epileptic children, leading to an FDA warning about monitoring patients on topiramate in case of hot weather. We present here the case of a topiramate-associated heatstroke in an adult patient suffering from essential tremor.

## Case presentation

A 57-year-old Caucasian man was admitted to the emergency room in a febrile comatose state. He was working as a chimney sweep and was known for hypertension, type II diabetes, dyslipidemia and essential tremor for which he had been prescribed topiramate 6 weeks earlier, at an initial dosage of 25 mg per day. The dosage was progressively increased by his physician, up to 50 mg twice a day on the day of admission. Other medication included citalopram, clonazepam, allopurinol, ibuprofen, mefenamic acid, metformin, esomeprazole, propranolol, losartan, torasemide, and hydrochlorothiazide.

Our patient was admitted in July, and the temperature on the day of his admission reached 39.7 °C (103.4 °F, heat index >93). The mean temperature of the week before was 36.6 °C (97.3 °F), making this week the second warmest week noted in Switzerland for more than 150 years, according to the national meteorological station [2].

Earlier on that day, as he was walking toward his car after work, our patient suddenly lost consciousness. At the arrival of the ambulance, our patient was found in a comatose state, with a Glasgow Coma Score (GCS) of 3. He was tachypneic with a rate of 50 breaths per minute with a preserved oxygen saturation and his tympanic temperature was more than 41 °C (105.8 °F), reaching the maximal scale of the thermometer. His blood pressure was 110/62 mmHg and his heart rate was 89 beats per minute. The onsite glycemia was 10 mmol/L. He was intubated and then brought to the emergency room. Given these circumstances, the initial neurologic examination was limited, but showed no asymmetrical pattern, no clonus, and no hyperreflexia.

On arrival, his temperature was 40.4 °C (104.7 °F) and he became hemodynamically shocked, needing volume resuscitation with 4 liters of sodium chloride 0.9 % and administration of noradrenaline. The electrocardiogram showed ventricular bigeminy and he received intravenous magnesium. Ceftriaxone and acyclovir were administrated, given a primary hypothetical diagnosis of meningoencephalitis.

Table 1 depicts the blood analyses at admission and a few hours later. They reflect a progressive multiple organ failure developing within 48 hours. A brain and thoracoabdominal computed tomography (CT) scan with injection of radiocontrast media showed no abnormalities except nonspecific, basilar pulmonary infiltrates of limited interest to explain our patient’s condition. Echocardiography showed a normal left ventricular ejection fraction and no pericardial effusion.

**Table 1:** Laboratory values at admission and worst values within 48 hours

Test	Unit	Value at admission	Worst value within 48 h	Value one week later
Hemoglobin	g/L	101	101	128
Leukocytes	G/L	20.6	20.6	7.3
Thrombocytes	G/L	181	131	377
PTT	seconds	>140	>140	25.5
INR		1.05	1.04	0.9
CRP	mg/L	2.5	6.4	5.1
Creatinine	umol/L	151	91	76
CK	U/L	160	829	50
AST	U/L	30	137	17
ALT	U/L	34	346	64
Alkaline phosphatase	U/L	39	48	72
GGT	U/L	122	183	112
Total bilirubin	umol/L	4	9	6

*D* day, *PTT* partial thromboplastin time, *INR* international normalized ratio, *CRP* C-reactive protein, *CK* creatine kinase, *AST* aspartate aminotransferase, *ALT* alanine transaminase, GGT gamma-glutamyl transpeptidase

A lumbar puncture showed an albuminocytologic dissociation (1 M/L leukocytes, 0.72 g/L proteins) without oligoclonal distribution, and an augmented glycorrachia (5 mmol/L). Cultures of the cerebrospinal fluid (CSF) remained sterile and viral polymerase chain reaction (PCR) assays [enteroviruses, human immunodeficiency virus (HIV), herpes simplex virus (HSV), human herpes virus 6 (HHV6), Epstein-Barr virus (EBV)] were negative, as well as blood serologies (HIV, parvovirus B19) and blood cultures. Toxicological analysis was only positive for benzodiazepines. The electroencephalogram (performed when our patient was already extubated and alert) showed no sign of epilepsy.

The hemodynamical and neurological courses were quickly favorable, allowing for an extubation and a transfer to a regular hospital ward within 24 hours. A clinical examination after extubation was strictly normal, including complete neurological status, and our patient was symptom-free.

Our patient could not recall what happened on the day he lost consciousness. Given our suspicion of heatstroke and the lack of evidence for an infectious disease, antibiotics and antivirals were stopped. His history was completed. Our patient had spent several hours in an unusually hot environment 3 days before the admission, while attending a meeting under a tent. It has to be pointed out that the weather had been exceptionally hot in Switzerland during this period of time, with temperatures reaching record levels. When specifically asked about his sweating, our patient immediately reported a decrease in his sweating since he started to take topiramate, while he used to sweat profusely before.

The clinical and biological courses were uneventful, with a normalization of all the parameters within 1 week, except for his gamma-glutamyl transpeptidase (GGT) level, which remained at the upper limits of normal, as already noted by his physician in the past.

## Discussion

Heatstroke is a life-threatening condition resulting in failure of normal compensatory heat-shedding mechanisms. When heat gain exceeds heat loss, the body temperature rises and can lead to heatstroke, defined as a body temperature higher than 40 °C (104 °F) associated with neurological dysfunction, ranging from mild altered mental status to coma [1]. Two forms of heatstroke can be distinguished: exertional and classical. Given the lack of evidence of strenuous exercise in our patient, although his profession could imply some physical efforts, classical heatstroke seems to better correspond to his condition: high temperature, coma, coagulation disorder, rhabdomyolysis, hepatic injury, and renal failure.

Topiramate is an antiepileptic agent with a broad spectrum of mechanisms of action, since blockage of voltage-dependent sodium channels, augmentation of gamma-aminobutyrate acid activity, antagonism of AMPA/kainate receptors, and inhibition of the carbonic anhydrase enzyme are known to contribute to its clinical efficacy [3]. Topiramate is indicated to treat certain types of seizures and to prevent migraine headaches [4]. Moreover, topiramate has also been recently designated as a first-line agent in the treatment of essential tremor [5]. The usual side effects of topiramate are nausea, fatigue, weight loss and paresthesia, but case reports in epileptic children have raised concerns about the risk of hypohidrosis and heatstroke [6–8].

The postulated mechanism of topiramate-associated hypohidrosis is thought to be related to an inhibition of the carbonic anhydrase enzyme within the sweat glands, leading to an impaired sweat composition with a reduced water formation and an impaired sweat rate. This hypothesis is supported by small electrophysiological studies showing that autonomic functions are normal in children and adults presenting topiramate-associated hypohidrosis [9, 10]. Moreover, research in mice suggested that topiramate reduces aquaporin expression in sweat glands.

Most of the hypohidrosis cases reported among children taking topiramate showed a good clinical evolution when the drug was stopped [6, 7]. Regarding adults, we found only six cases of patients diagnosed with topiramate-related heatstroke, with unfavorable outcome for half of them (one death and two with persistent neurological dysfunction) [11–13]. The other half, as well as our patient, completely recovered [14]. Doses of topiramate ranged between 200 and 600 mg/day. Four patients were treated for seizures, one for migraine and one for aggressive behavior. In a prospective, controlled cohort study, 10.5 % of patients on topiramate spontaneously reported hyperthermia when they were asked about side effects in general compared to 0.5 % of patients on other antiepileptics [15]. Described risk factors to develop topiramate-associated hyperthermia are young age, high doses and hot weather [15].

In our patient’s case, the dosage of topiramate was rather low, but our patient was taking ibuprofen, mefenamic acid, losartan and diuretics, which probably contributed to acute renal failure in this diabetic patient, potentially leading to an accumulation of topiramate, since this drug is eliminated by the kidney. Unfortunately, topiramate blood levels were not requested at admission. They were low (9.9 umol/L, normal 15–80 umol/L) 2 days later, after decreasing the dosage from 50 mg twice a day to 50 mg once a day. Second, the weather on the day of admission and the past few days had been exceptionally hot, reaching 39.7 °C (103.4 °F) on the day of admission.

Regarding the results of the lumbar puncture, there are no clear data describing how the CSF composition should evolve during a heatstroke. Among the six other adult patients diagnosed with topiramate-related heatstroke, four of them had had a lumbar puncture [11, 14] which was described as normal and sterile. The albuminocytologic dissociation we found may be explained by the inflammatory state, leading to an enhanced permeability of the membranes and an exudation of proteins into the CSF, as would be observed during systemic inflammation response syndrome (SIRS).

Our patient had a cerebral CT scan described as normal, which coincides with the other cases reported [11, 14]. Only one patient with persistent neurological dysfunction benefited from a cerebral magnetic resonance imaging (MRI) scan [11], which showed diffuse bilateral lesions, disappearing with time. However, a diffuse atrophy persisted, suggesting irreversible neural damage. We did not perform an MRI scan on our patient given his complete neurological recovery.

Since our patient was taking a selective serotonin reuptake inhibitor at a therapeutic dosage on the day of admission, the clinical manifestations (encephalopathy and hyperthermia) could have raised the suspicion of serotonin syndrome. Our patient was in a comatose state and needed to be quickly intubated, therefore the initial neurological examination was restricted, but clonus and hyperreflexia were not specifically documented. Serotonin syndrome was ruled out because citalopram had always been well tolerated before, and its dosage had remained unchanged over the past few weeks before admission. In addition, citalopram is partly metabolized by the cytochrome P450 CYP3A4, which is induced by topiramate. Given this mechanism, the recent introduction of topiramate would have theoretically led to a decrease of citalopram plasma concentration, thus further reducing the likelihood of serotonin syndrome. Moreover, the encephalopathy and hyperthermia resolved despite citalopram continuation.

## Conclusions

In conclusion, we report the case of a heatstroke in the context of topiramate treatment in an adult patient, with complete recovery. Unlike other case reports of adult patients with acute, severe outcomes, we were able to document CSF characteristics and perform an extensive infection workup, thus increasing the likelihood of the diagnosis and providing CSF particularities in this condition.

Topiramate-related heatstroke has particularly been described in epileptic children but must be as well recognized in adults treated for migraine and essential tremor, with lower dosages. Outcomes may range from total clinical recovery to persistent neurological dysfunction or death. The prescription of topiramate and the follow-up of adult patients under this medication should include an evaluation of hypohidrosis and hyperthermia especially in contexts of high external temperature.

## Consent

Written informed consent was obtained from the patient for publication of this case report. A copy of the written consent is available for review by the Editor-in-Chief of this journal.

## Abbreviations

CSF: Cerebrospinal fluid
CT: Computed tomography
EBV: Epstein-Barr virus
GCS: Glasgow Coma Scale
GGT: Gamma-glutamyl transpeptidase
HHV6: Human herpes virus 6
HIV: Human immunodeficiency virus
HSV: Herpes simplex virus
MRI: Magnetic resonance imaging
PCR: Polymerase chain reaction
SIRS: Systemic inflammation response syndrome
